# Flavonol and imidazole derivatives block HPV16 E6 activities and reactivate apoptotic pathways in HPV^+^ cells

**DOI:** 10.1038/cddis.2015.391

**Published:** 2016-01-21

**Authors:** C-H Yuan, M Filippova, J L Krstenansky, P J Duerksen-Hughes

**Affiliations:** 1 Department of Basic Sciences, Loma Linda University School of Medicine, 11021 Campus Street, 101 Alumni Hall, Loma Linda, CA 92354, USA; 2 KGI School of Pharmacy, 535 Watson Drive, Claremont, CA 91711, USA

## Abstract

High-risk human papillomaviruses (HR-HPVs) cause nearly all cases of cervical cancer, as well as approximately 30% of head and neck cancers. HPV 16 E6, one of two major viral oncogenes, protects cells from apoptosis by binding to and accelerating the degradation of several proteins important in apoptotic signaling, including caspase 8 and p53. We proposed that blocking the interactions between HPV E6 and its partners using small molecules had the potential to re-sensitize HPV^+^ cells to apoptosis. To test this idea, we screened libraries of small molecules for candidates that could block E6/caspase 8 binding and identified several candidates from different chemical classes. We tested hits for dose-dependency and specificity *in vitro* and for toxicity in a cell-based assay and then used this information to select the two best candidates for further testing: myricetin, a flavonol, and spinacine, an imidazole amino-acid derivative of histidine. Both compounds clearly inhibited the ability of E6 to bind *in vitro* to both caspase 8 and E6AP, the protein that mediates p53 degradation. In addition, both compounds were able to increase the level of caspase 8 and p53 in SiHa cervical cancer cells, resulting in an increase of caspase 3/7 activity. Finally, both myricetin and spinacine sensitized HPV^+^ cervical and oral cancer cells, but not HPV^−^ cervical and oral cancer cells, to apoptosis induced by the cancer-specific ligand TRAIL, as well as the chemotherapeutic agents doxorubicin and cisplatin. New therapies based on this work may improve treatment for HPV^+^ cancer patients.

High-risk types of human papillomavirus (HPV), especially types 16 and 18, are the causative agents of nearly all cases of human cervical cancer, in addition to up to 70% of head and neck cancers (HNC).^1^ Although the overall incidence of HNC has stabilized during the past decade, the incidence of HPV-associated cases, especially of oropharyngeal squamous cell carcinoma, has dramatically increased.^2^ HPVs are small, double-stranded DNA viruses that infect epithelial tissues. The HPV-encoded oncogenes E6 and E7 are responsible for cellular immortalization and transformation and, consequently, for the development of HPV-associated cancer. Although E7 is best known for the inactivation of Rb, E6 accelerates the degradation of several molecules involved in apoptosis.

Two HPV vaccines, Gardasil (MSD, Merck, Kenilworth, NJ, USA) and Cervarix (GSK, Glaxo SmithKline, London, UK), have been approved and are currently in use for the prevention of HPV infection. However, they offer no benefit to an individual who has already been infected and only protect against 2 of the 15 types of high-risk viruses, HPV-16 and -18. Surgical and ablative techniques are used to remove developed tumors; however, these approaches are invasive and cytodestructive, and lesions frequently recur following treatment. Chemotherapy, utilizing agents such as cisplatin and doxorubicin, has also been used to treat cervical cancer but with mixed results.^3, 4, 5, 6, 7, 8^ As researchers and clinicians have worked to move beyond these relatively non-specific and toxic agents, reagents that activate the tumor necrosis factor-related apoptosis-inducing ligand (TRAIL)-mediated, extrinsic apoptotic pathway have garnered considerable interest owing to their promise in the treatment of several types of tumors.^9, 10, 11, 12, 13, 14, 15, 16, 17^

Unfortunately, therapies that function by activating apoptosis, including those based on TRAIL, cisplatin and doxorubicin, are handicapped in their ability to effectively treat HPV-associated malignancies, because high-risk E6 proteins subvert both the extrinsic and intrinsic apoptotic pathways. E6 proteins from high-risk types of HPV are well known for their ability to mediate the rapid degradation of p53,^18, 19, 20, 21^ an important mediator of intrinsic apoptotic pathways, thereby increasing the growth and survival of transformed cells.^22, 23^ E6 also interacts with other partner proteins, a number of which participate in extrinsic, receptor-mediated apoptosis. For example, our laboratory found that HPV 16 E6 binds to and inactivates several molecules involved in these pathways, including TNF R1,^24^ Fas-associated protein with death domain (FADD),^25^ and caspase 8.^26, 27^ As a result, engagement of either the extrinsic or the intrinsic apoptotic pathways fails to result in the transduction of the intended death signal because the mediator molecules – p53 in the case of the intrinsic pathway, and FADD and caspase 8 in the case of the extrinsic pathway – are missing. Therefore, if any of these apoptosis-inducing signaling pathways are to be used as effective tools for the elimination of HPV-associated malignancies, it will be necessary to restore the missing signaling molecules.

In our previous work,^28^ we identified myricetin as a compound that can inhibit the E6/caspase 8 interaction *in vitro*. Unfortunately, myricetin is known to also inhibit a number of cellular proteins, including several tyrosine kinases, and its structure makes modification for drug development difficult. The identification of additional, more tractable inhibitors of the E6/procaspase 8 interaction was therefore pursued. Screening an ActiProbe 2 K library enabled us to identify several compounds of interest, and we further examined the ability of one particular compound, spinacine (**5**; see Figure 1), an unnatural amino acid that is the product of the action of formaldehyde on histidine, to inhibit specific interactions between E6 and its partner proteins. We found that both myricetin and spinacine can re-sensitize HPV^+^ cells to apoptosis triggered by apoptotic inducers such as TRAIL, cisplatin, and doxorubicin by increasing caspase 3/7 activity and restoring the level of apoptotic proteins in HPV^+^ cells. These findings identify a novel small molecule that can inhibit E6 functions and may serve as the basis for further discovery and development of effective and novel therapeutic approaches for the treatment of HPV-mediated cancers.

## Results

### Imidazole derivatives specifically inhibit the interaction of HPV E6 with caspase 8

**Figure 1:**
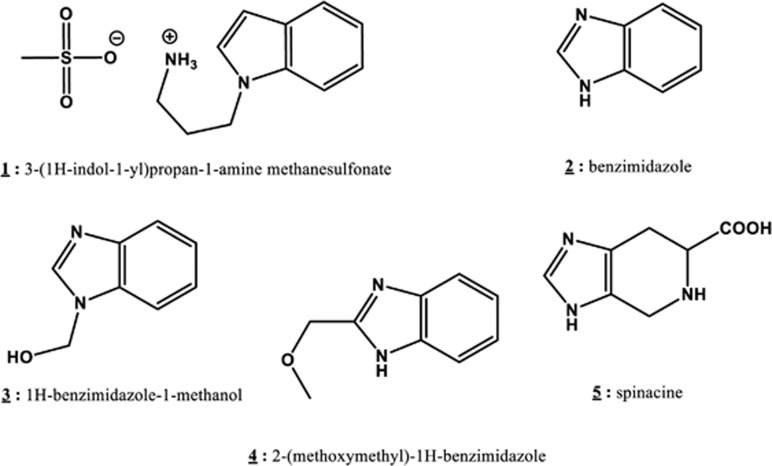
Structures of the tested potential E6 ligands

We previously reported that HPV E6 binds to caspase 8^29^ and that myricetin can block the E6/caspase 8 interaction *in vitro*.^28^ In that study, we utilized a bead-based assay based on AlphaScreen Technology (Perkin-Elmer, Waltham, MA, USA) to identify inhibitors of the E6/caspase 8 interaction.^28^ In the current work, we extended our search by screening the ActiProbe 2 K (2000 compounds) library using the same approach. In our primary screen, 118 (5.9%) compounds demonstrated an ability to inhibit the E6/caspase 8 interaction. Seventy-nine of these 118 compounds presented EC_50_ values <10 *μ*M and were therefore chosen for further analysis. Twenty-three of these 79 compounds also demonstrated specific inhibition of the E6/caspase 8 interaction, in that they were unable to block formation of the caspase 8/caspase 8 homodimer (counter-screen). These 23 compounds represented three different chemical classes, of which the imidazoles and the related benzimidazoles showed the best inhibition of E6/caspase 8 binding. Analysis of the structures and physical/chemical characteristics of those molecules led to the selection of five additional compounds: 3-(1H-indol-1-yl)propan-1-amine methanesulfonate (**1**), benzimidazole (**2**), 1H-benzimidazole-1-methanol (**3**), 2-(methoxymethyl)-1H-benzimidazole (**4**), and spinacine (**5**) for further study (Figure 1).

**Figure 2:**
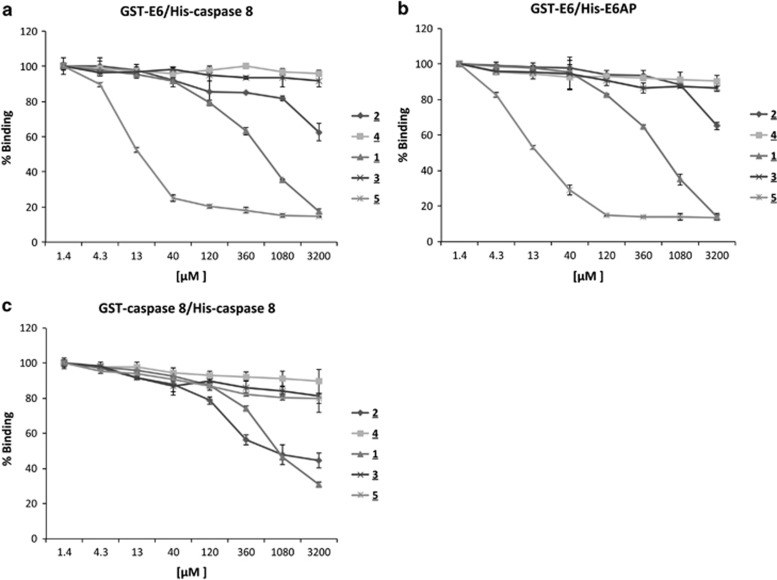
3-(1H-indol-1yl)propan-1-amine (**1**), three benzimidazole derivatives (**2**–**4**), and spinacine (**5**) inhibit protein–protein interactions. Five compounds at the indicated concentrations (1.4 *μ*M–3.2 mM) were tested using our bead-based screening assay for their ability to inhibit three different protein/protein interactions: (**a**) GST-E6/His-caspase 8; (**b**) GST-E6/HisE6AP; and (**c**) GST-caspase 8/His-caspase 8. Binding in the presence of 1.4 *μ*M of the test compound was set at 100%. Experiments were performed in triplicate, and error bars indicate the S.D.

To determine the dose-responsiveness of these five compounds, variable concentrations of those small molecules were first tested for their ability to inhibit E6/caspase binding. The results showed that only compounds **1**, **2**, and **5** demonstrated dose-dependent inhibition of E6/caspase 8 binding (Figure 2a). We then asked whether these molecules, which had been selected for their ability to interfere with E6/caspase 8 binding, could also affect other interactions of E6, such as its binding to E6-associated protein (E6AP). The E6/E6AP interaction is required for the E6-mediated acceleration of p53 degradation.^21^ We therefore tested the ability of these five compounds to inhibit E6/E6AP binding and found that compounds **1**, **2**, and **5**, the same agents that had inhibited E6/caspase 8 binding, could also inhibit the E6/E6AP interaction and could do so nearly as well as they had inhibited the E6/caspase 8 interaction (Figures 2a and b).

To ask whether this inhibition was specific, we employed a counter-screening assay in which we assessed the ability of candidates to inhibit the binding of GST-caspase 8 to His-caspase 8; those that did were to be eliminated. In this counter-screening assay, the materials in each well were the same as in the primary screen, with the exception that GST-caspase 8 replaced GST-E6. Compounds **1** and **2** were able to inhibit the caspase 8/caspase 8 interaction about as well as they inhibited the E6/caspase 8 interaction, indicating that their actions were non-specific. However, spinacine (**5**) did not significantly inhibit the binding of caspase 8 to itself, providing evidence of its specificity for E6 (Figure 2c). These results are summarized in Table 1, which shows that, of the compounds tested, spinacine displayed the lowest EC_50_ value for the inhibition of E6/caspase 8 and E6/E6AP interactions, while not inhibiting caspase 8/caspase 8 binding.

### Myricetin and spinacine sensitize SiHa cells to TRAIL-induced apoptosis

**Figure 3:**
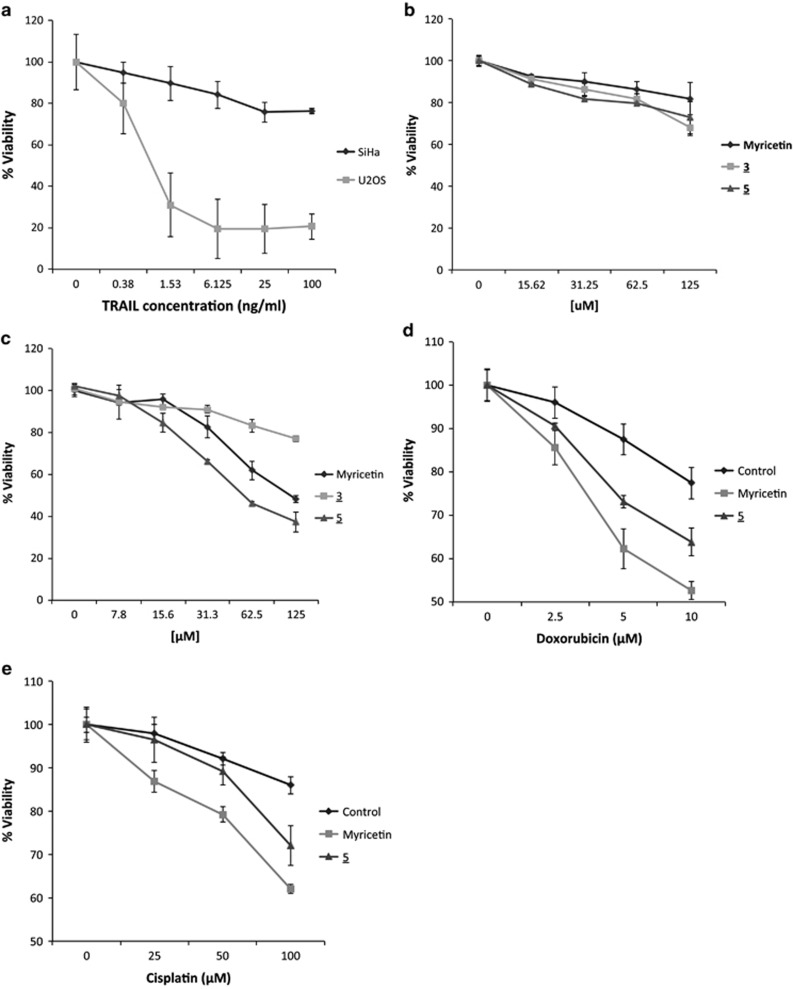
(**a**) HPV^+^ SiHa cells are resistant to TRAIL-induced apoptosis as compared with the human osteosarcoma cell line U2OS. Cells (2 × 10^4^ cells per well) were seeded into a 96-well plate, allowed to incubate overnight, and then treated with the indicated concentration of TRAIL in the presence of cycloheximide (5 *μ*g/ml). The viability of cells was measured after overnight incubation using the MTT assay; the viability of cells untreated with TRAIL was set at 100% for each group. (**b**) The indicated concentrations of three small molecules were added to SiHa cells, and an MTT assay was preformed after overnight incubation. The viability of cells untreated with small molecules was set as 100%. (**c**) The indicated concentrations of myricetin or spinacine were added 4 h prior to TRAIL (100 ng/ml) in the presence of cycloheximide (5 *μ*g/ml) and incubated at 37 °C overnight. The viability of cells untreated with small molecules was set at 100%. (**d** and **e**) SiHa cells (2 × 10^4^ cells per well) were seeded into a 96-well plate and allowed to incubate overnight. In all, 100 *μ*M of myricetin and 50 *μ*M of spinacine were added 4 h prior to either (**d**) doxorubicin or (**e**) cisplatin. Cell viability was measured after overnight incubation using the MTT assay, and the viability of cells untreated with either myricetin or spinacine was set at 100%. Experiments were performed in triplicate, and error bars indicate the S.D.

We next asked which of the molecules that block E6/caspase 8 binding *in vitro* could also act in the context of a cell. SiHa cells are an HPV^+^ cell line, derived from a cervical carcinoma, which serves as a commonly used model for HPV-associated malignancies. To determine whether HPV^+^ SiHa cells are resistant to TRAIL-induced apoptosis, SiHa cells were treated with TRAIL and cell viability was assessed. TRAIL-sensitive, HPV^−^ U2OS cells served as a positive control. The results (Figure 3a) demonstrate that, in comparison to U2OS cells, SiHa cells are relatively resistant to treatment with TRAIL, as predicted. Furthermore, both myricetin and spinacine displayed low toxicity to SiHa cells in the absence of TRAIL (Figure 3b). We next asked whether myricetin and/or spinacine could sensitize these HPV^+^ cells to TRAIL-induced apoptosis. The indicated concentrations (0 –125 *μ*M) of myricetin, (**3**) (negative control), and spinacine (**5**) were added to SiHa cells in the presence of TRAIL, and Figure 3c shows that, in the presence of TRAIL, myricetin and spinacine were both able to reduce the viability of SiHa cells to 30–40% at a dose of 125 *μ*M, in contrast to the negative control, **3**. Table 2 lists and compares the concentrations of the three tested compounds required to induce death in 50% of SiHa cells in the presence or absence of TRAIL and demonstrates that both myricetin and spinacine induced more cell death (an increase of 3.5-fold for myricetin and of 8.6-fold for spinacine) in the presence than in the absence of TRAIL (Table 2). These results provide strong evidence that inhibiting the E6/caspase 8 interaction can restore the sensitivity of HPV^+^ cells to apoptotic signals.

### Myricetin and spinacine increase the sensitivity of SiHa cells to doxorubicin and cisplatin

As noted earlier, both myricetin and spinacine can also inhibit the binding of E6 to E6AP, an E3 ligase involved in the degradation of p53, *in vitro*. Based on this observation, we next asked whether myricetin and/or spinacine would increase the sensitivity of SiHa cells to two drugs, doxorubicin and cisplatin, that are thought to act by inducing intrinsic apoptosis through the activation of pathways that involve p53. We found that, as compared with the negative control, both myricetin and spinacine increased the sensitivity of SiHa cells to doxorubicin and cisplatin (Figures 3d and e). These results suggest that the ability of both myricetin and spinacine to inhibit the interaction between E6 and E6AP, resulting in a predicted increase in p53, results in the sensitization of HPV^+^ cells to inducers of p53-mediated apoptosis.

### Myricetin and spinacine increase caspase 3/7 activity

**Figure 4:**
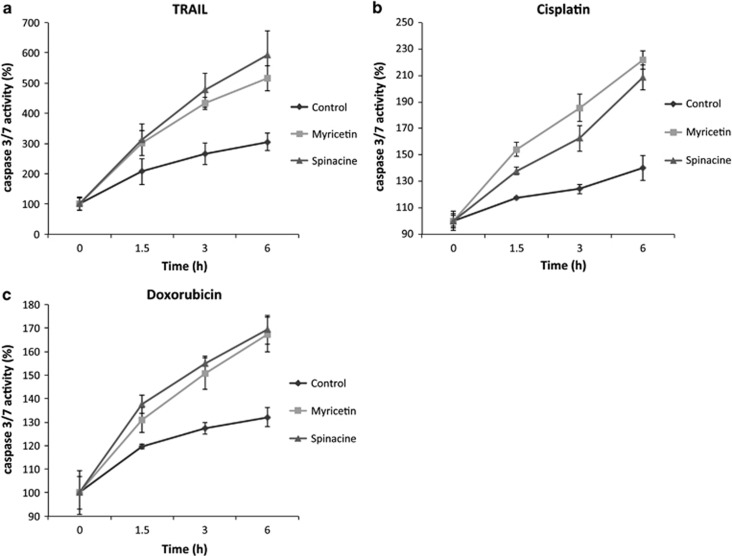
Myricetin and spinacine (**5**) increase caspase 3/7 activity in SiHa cells following treatment with TRAIL and chemotherapy drugs. SiHa cells (2 × 10^4^ cells per well) were seeded into a 96-well plate and allowed to incubate overnight and then pretreated with 100 *μ*M of myricetin or 50 *μ*M of spinacine for 4 h. (**a**) TRAIL (100 ng/ml), along with cycloheximide (5 *μ*g/ml), (**b**) cisplatin (50 *μ*M), or (**c**) doxorubicin (2 *μ*M) were added respectively. Caspase 3/7 activity was measured after 0, 1.5, 3, and 6 h using the CellTiter-Glo assay. Activity at 0 h of treatment was set at 100% for each group. Experiments were performed in triplicate, and error bars indicate the S.D.

Activation of caspase 3/7 is an essential marker for both extrinsic and intrinsic apoptosis and can be used to determine whether cell death occurs through the apoptotic pathway. Our previous data (Figure 2) demonstrates that both myricetin and spinacine can inhibit the E6/caspase 8 and E6/E6AP interactions *in vitro*, resulting in increased cell death. If this cell death occurred through apoptosis, one prediction is that pretreatment of cells with either myricetin or spinacine should increase the level of TRAIL-, cisplatin-, and doxorubicin-induced activation of caspase 3/7. To test this prediction, caspase 3/7 activity was measured in SiHa cells following TRAIL, cisplatin, and doxorubicin treatment in the presence or absence of myricetin or spinacine. As shown in Figure 4a, we found that the caspase 3/7 activity increased when myricetin or spinacine were combined with TRAIL as compared with controls. Similar results were obtained following treatment with cisplatin and doxorubicin (Figures 4b and c). These data demonstrate that the increased cell death induced by the combination of apoptotic inducers and myricetin/spinacine occurs through the apoptotic pathway.

### Myricetin and spinacine re-sensitize HPV^+^ cells to TRAIL-induced apoptosis by blocking the binding of HPV E6 to caspase 8

**Figure 5:**
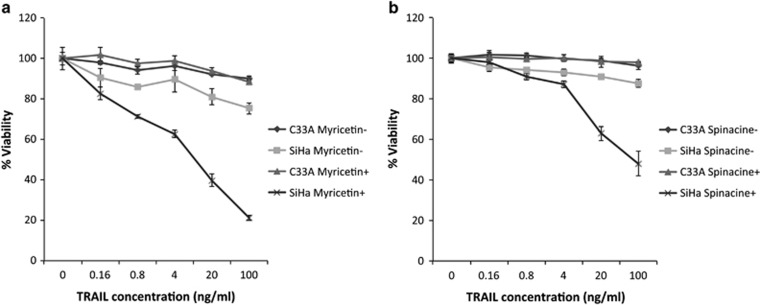
Myricetin and spinacine (**5**) can re-sensitize HPV^+^, but not HPV^−^, cells to treatment with TRAIL. SiHa (2 × 10^4^ cells per well) and C33A cells (1 × 10^4^ cells per well) were seeded into a 96-well plate and allowed to incubate overnight, and then cells were pretreated in the presence or absence of (**a**) myricetin (100 *μ*M) or (**b**) spinacine (50 *μ*M) for 4 h. The indicated concentration of TRAIL along with cycloheximide (5 *μ*g/ml) was added, and the cells were allowed to incubate overnight. Cell viability was measured by MTT assay, and the viability of cells untreated with TRAIL was set at 100%. Experiments were performed in triplicate, and error bars indicate the S.D.

To determine whether myricetin and spinacine re-sensitize SiHa cells to TRAIL-induced apoptosis by specifically blocking the binding of E6 to caspase 8, we compared responses in the HPV^+^ SiHa cells with those from the HPV^−^ human cervical carcinoma cell, C33A, as these cells do not express E6. We found that, although the HPV^+^ SiHa cells displayed resistance to TRAIL at concentrations up to 100 ng/ml, their sensitivity increased dramatically in the presence of 100 *μ*M myricetin. In contrast, the HPV^−^ C33A cells remained resistant to TRAIL-mediated apoptosis even in the presence of myricetin (Figure 5a). Similar results were also found following pretreatment with 50 *μ*M spinacine (Figure 5b). Together, these results suggest that myricetin and spinacine can re-sensitize HPV^+^ cells to TRAIL-mediated apoptosis by specifically blocking the binding of HPV E6 to apoptotic proteins, such as caspase 8.

### Myricetin and spninacine increase the levels of caspase 8 and p53 in SiHa cells

**Figure 6:**
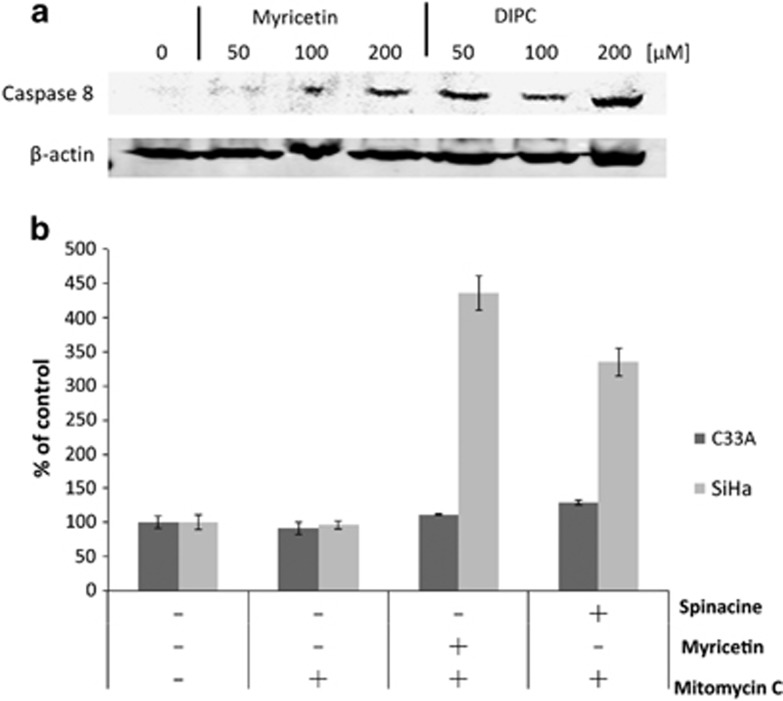
Treatment with myricetin and spinacine (**5**) increases cellular levels of caspase 8 and p53. (**a**) SiHa cells (1 × 10^6^ per well) were seeded into a six-well plate and allowed to incubate overnight. The indicated concentrations of myricetin and spinacine were added, and then cells were incubated for 24 h. Cells were then washed in 1 × PBS and then harvested, and the resulting level of caspase 8 was measured by immunoblot. (**b**) SiHa and C33A cells (1 × 10^6^ per well) were seeded into a six-well plate and allowed to incubate overnight. In all, 200 *μ*M of myricetin and 100 *μ*M spinacine were added together with 4 *μ*g/ml mitomycin C and incubated for 24 h. The resulting level of p53 was measured by enzyme-linked immunosorbent assay, and the level of p53 found in untreated cells was set at 100%. Experiments were performed in triplicate, and error bars indicate the S.D.

The data described above demonstrate that myricetin and spinacine are able to increase caspase 3/7 activity (Figure 4) and to sensitize SiHa cells to TRAIL (Figures 3a and c and 5) and p53 (Figures 3d and e) induced apoptosis. If this ability is due to inhibition of E6 functions, then an increase in the levels of caspase 8 and p53 should be observable in SiHa cells following treatment with myricetin and spinacine. To test this prediction, we treated SiHa cells with 50, 100, and 200 *μ*M of myricetin or spinacine for 24 h and then measured the level of caspase 8 by immunoblot. The results (Figure 6a) demonstrate that treatment with myricetin caused an increase in caspase 8 levels at doses of 100 and 200 *μ*M, while spinacine caused an increase in caspase 8 at doses from 50 to 200 *μ*M. To ask how p53 levels were affected, we employed an ELISA assay. For comparison, C33A cells, which lack HPV E6 and express mutant p53, were also treated with the same concentrations of spinacine and myricetin. Mitomycin C was included in the assay to cause DNA damage and therefore increase p53 levels. The results from this experiment (Figure 6b) demonstrate that treatment of SiHa cells with myricetin caused a 4.4-fold increase in p53 at a dose of 200 *μ*M and that treatment with spinacine caused a 3.4-fold increase in p53 at a dose of 100 *μ*M, as compared with untreated cells. Furthermore, there was no significant change in p53 levels in C33A cells, confirming our earlier conclusion that myricetin and spinacine impact apoptotic pathways only in the presence of E6. Together, these results suggest that these small molecules are able to increase the sensitivity of SiHa cells to both TRAIL- and p53-induced apoptosis by blocking the ability of E6 to bind to its cellular partners, thereby reducing the degradation of caspase 8 and p53 in HPV^+^ SiHa cells.

### Myricetin and spinacine sensitize HPV^+^ head and neck squamous cell carcinoma (HNSCC) cells to TRAIL-induced cell death

**Figure 7:**
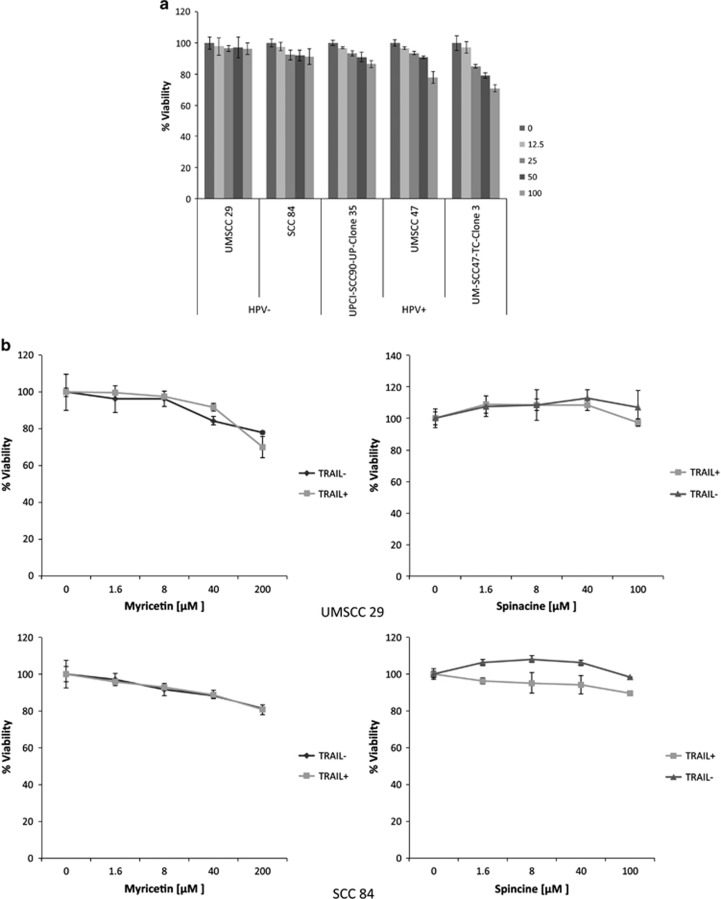

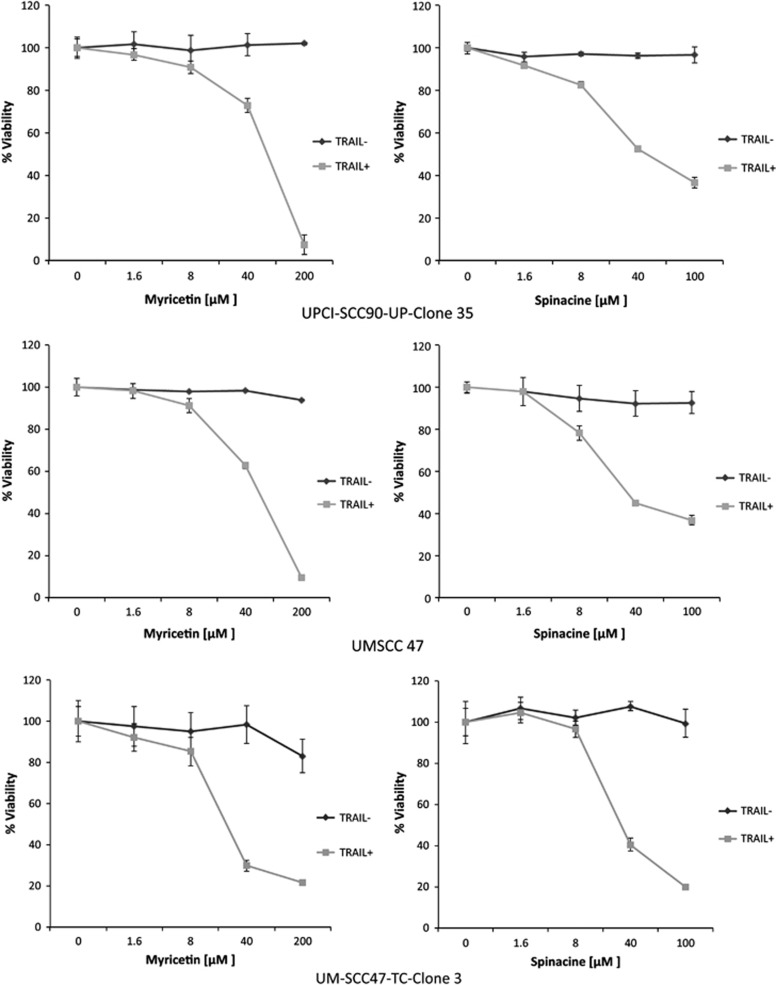
Myricetin and spinacine (**5**) can re-sensitize HPV^+^, but not HPV^−^, head and neck cancer cell lines to treatment with TRAIL. (**a**) Both HPV^−^ and HPV^+^ HN cancer cell lines display resistance to TRAIL treatment. (**b**) HPV^−^ (UMSCC 29 and SCC 84) and (**c**) HPV^+^ (UMSCC 47, UPCI-SCC90-UP-Clone 35, and UM-SCC47-TC-Clone 3) head and neck cancer cell lines (2 × 10^4^ cells per well) were seeded into 96-well plates and allowed to incubate overnight, and then cells were pretreated with myricetin (0–200 *μ*M) or spinacine (0–100 *μ*M) for 4 h. In all, 50 *μ*M of TRAIL along with cycloheximide (5 *μ*g/ml) was added, and cells were allowed to incubate overnight. Cell viability was measured by the MTT assay, and the viability of cells untreated with small molecules was set at 100%. Experiments were performed in triplicate, and error bars indicate the S.D.

As shown above, myricetin and spinacine sensitize SiHa cells to TRAIL and chemotherapy drugs. These data provide proof-of-principle that small-molecule inhibitors can block HPV E6 functions and trigger HPV^+^ cervical cancer cell lines to undergo apoptosis. We next examined whether other types of HPV^+^ cancer cells, and in particular, HNSCC cells can also be sensitized in this manner. All selected cell lines demonstrated resistance to TRAIL at concentrations as high as 100 ng/ml (Figure 7a). For the combination experiments, 50 ng/ml TRAIL was applied in combination with either myricetin (0–200 *μ*M) or spinacine (0–100 *μ*M). As shown in Figure 7b, the HPV^−^ HNSCC cell lines (UMSCC29, #29; and SCC84, #84) were resistant to TRAIL in both the presence and absence of myricetin or spinacine. In contrast (Figure 7c), TRAIL-induced cell death was enhanced 40% by 40 *μ*M myricetin in the HPV^+^ cell line UMSCC47, #47; by 30% in UPCI-SCC90-UP-Clone 35, #90; and by 70% in the UM-SCC47-TC-Clone 3, 47CL3. Furthermore, 40 *μ*M spinacine was able to enhance TRAIL-induced cell death in the HPV^+^ #47 and #90 cell lines up to 50% and near 60% in the HPV^+^ #47CL3 cell line. Together, these results indicate that both myricetin and spinacine sensitize HPV^+^, but not HPV^−^, HNSCC cell lines to TRAIL-induced apoptosis, confirming our previous results that these small molecules block HPV E6 functions.

### Myricetin and spinacine sensitize HPV^+^ HNSCC cells to doxorubicin

**Figure 8:**
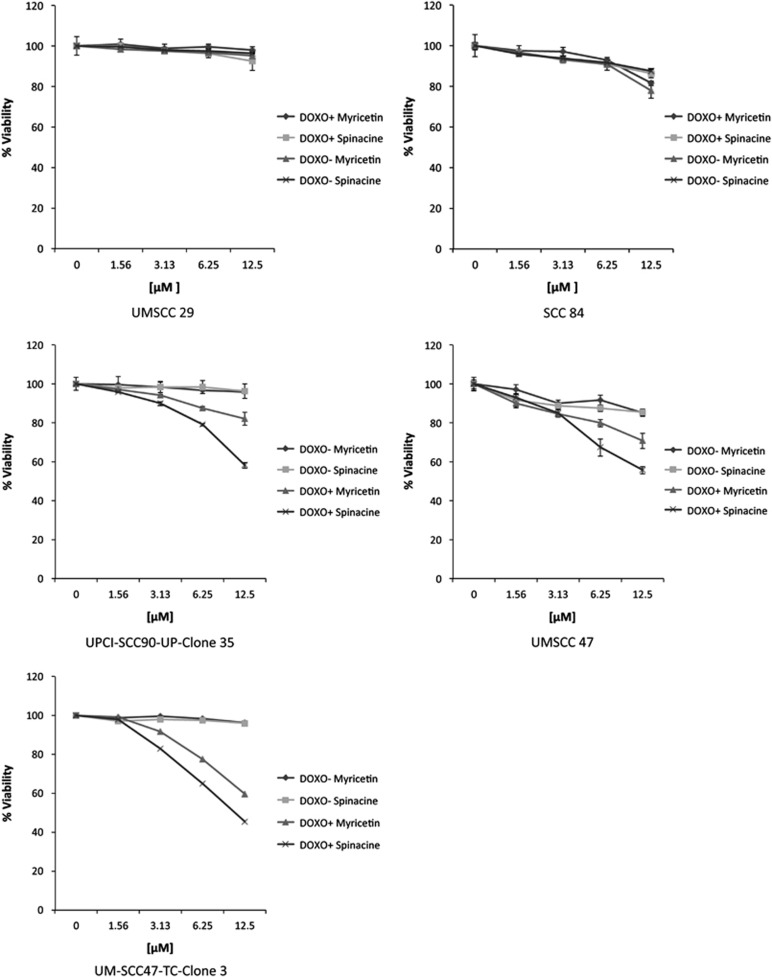
Myricetin and spinacine (**5**) can re-sensitize HPV^+^, but not HPV^−^, HNSCC cell lines to treatment with doxorubicin. Two HPV^−^ (UMMSCC 29 and SCC 84) and three HPV^+^ (UMSCC 47, UPCI-SCC90-UP-Clone 35, and UM-SCC47-TC Clone 3) head and neck cancer cell lines (2 × 10^4^ cells per well) were seeded into 96-well plates and allowed to incubate overnight, and then cells were pretreated with myricetin or spinacine (0–12.5 *μ*M) for 4 h. In all, 2 *μ*M of doxorubicin was added, and the cells were allowed to incubate overnight. Cell viability was measured by MTT assay, and the viability of cells untreated with small molecules was set at 100%. Experiments were performed in triplicate, and error bars indicate the S.D.

The results described above indicate that myricetin and spinacine sensitize HPV^+^ HNSCC cells to TRAIL-induced apoptosis. To ask whether this sensitization also occurs in the context of chemotherapeutic agents, several HNSCC cell lines were tested for their responses to combination treatments. As shown in Figure 8a, the HPV^−^ HNSCC cell lines UMSCC 29 (#29) and SCC 84 (#84) were resistant to doxorubicin in both the presence and absence of myricetin and spinacine. However, HPV^+^ HNSCC cancer cell lines, such as UPCI-SCC90-UP-Clone 35 (#90), UMSCC 47 (#47), and UM-SCC47-TC-Clone 3 (#47 CL3), displayed an increase in cell death when treated by combinations of 2 *μ*M doxorubicin plus the indicated small molecules (Figure 8b). In these experiments, spinacine demonstrated a higher efficiency than did myricetin, sensitizing #47, #47CL3, and #90 to doxorubicin by up to 50% at a concentration of 12.5 *μ*M. Together, these data demonstrate that myricetin and spinacine can sensitize both cervical and oral HPV^+^ cancer cells to chemotherapeutic agents such as doxorubicin.

## Discussion

Currently, no small molecules targeting any of the HPV proteins are available for clinical use, although a few groups are examining the possibility of developing these sorts of small, inhibitory molecules to expand and enhance the limited therapeutic options currently available for HPV-associated malignancies.^30, 31^ E6 and E7 oncogenes are expressed at relatively high levels during cancer development and therefore have the potential to serve as useful targets. For example, inhibition of E6 is predicted to lead to increased cell death, as E6 normally functions to block both intrinsic and extrinsic apoptotic pathways.^32^

One approach to blocking the interactions between E6 and its cellular partners is to use peptides, and previous work from our laboratory and others has identified peptide inhibitors that can specifically inhibit the interactions between E6 and caspase 8 and FADD^33^ or between E6 and E6AP.^33, 34^ As compared with peptide inhibitors, however, small molecules possess numerous advantages because they are more stable, penetrate target cells more easily, and can more readily be modified and optimized by organic chemists during drug development. Some progress has been made in this area, as published studies have identified several compounds (some with EC_50_ values between 17 and 29 *μ*M) that could inhibit the interaction between E6 and E6AP.^30, 35^ However, no structure–activity relationship (SAR) has been carried out for those compounds because of the limited data set, and none of these findings have yet led to clinically useful interventions.

Our laboratory previously reported that the flavonol myricetin can inhibit interactions between E6 and caspase 8 *in vitro*, and preliminary results (data not shown) indicate that myricetin binds to E6 at a ratio of 2 : 1. Unfortunately, myricetin is known to inhibit a number of cellular proteins (including several kinases), indicating a lack of sufficient specificity. In addition, the high EC_50_ value observed in the context of a cell-based model system (Table 2) suggests that its ability to negatively affect tumors may be limited, perhaps because its polar nature limits its ability to enter cells. As polar groups are an important feature in assessing SARs, optimization of this series of compounds would be difficult without resorting to a pro-drug approach. Therefore, we engaged in additional screening efforts with libraries containing more ‘lead-like' members that are expected to provide better platforms for optimization.

Of the five tested compounds (Figure 1), spinacine (**5**) was best able to specifically inhibit the interactions of E6 with both caspase 8 and E6AP (Figures 2a and b), demonstrating the lowest EC_50_ values for both E6/Caspase 8 and E6/E6AP binding. However, it did not inhibit caspase 8/caspase 8 binding, thus demonstrating the desired specificity (Table 1). In the experiments that involved treating SiHa cells with TRAIL, the EC_50_ values demonstrate that, as compared with myricetin, spinacine may be better able to penetrate into cells and thus re-sensitize cells more efficiently to TRAIL-induced apoptosis (Table 2).

Therapies based on TRAIL-mediated apoptosis, alone or in combination with other agents, are attractive possibilities for the treatment of many different types of cancer.^14, 15, 16, 17^ However, these treatments are unlikely to be helpful in the context of HPV on their own, owing to the ability of E6 to compromise apoptotic pathways. Our study found that the combination of TRAIL with small molecules can re-sensitize E6-expressing cells to TRAIL-induced apoptosis, potentially filling this gap. In addition to sensitizing cells to TRAIL, we found that both myricetin and spinacine sensitized cells to intrinsic apoptosis triggered by chemotherapy drugs, such as doxorubicin and cisplatin. This may be clinically relevant, because cervical cancer tends to be relatively resistant to chemotherapeutic treatments, such as cisplatin.^36^ We note that myricetin required a higher concentration (100 *μ*M) than did spinacine (50 *μ*M) to inhibit binding and to sensitize cells, suggesting that spinacine may be a more efficient agent. Taken together, these data suggest that myricetin and spinacine can block the binding of E6 to multiple apoptotic proteins, including caspase 8 and E6AP/p53, thereby reactivating the E6-compromised apoptotic pathways and rendering HPV^+^ cells sensitive to both intrinsic and extrinsic inducers of apoptosis.

The ability of myricetin and spinacine to sensitize HPV^+^ SiHa cells to apoptosis induced by TRAIL and chemotherapy agents led us to ask whether the same effects could be seen in HPV^+^ HNC cells. We found that the result from our HNC cells closely mirrored those from the cervical cells, indicating that our results should have broad applicability to HPV-associated malignancies regardless of the site of origin.

Our data suggest a mode of action in which the small molecule interacts directly with E6, either destabilizing the virus protein and changing its conformation, or blocking the interactions by direct interference. If the latter, one possibility is that myricetin/spinacine binds to a region on E6 required by both E6AP and caspase 8. Further work is necessary to develop a detailed model for the interactions between E6 and E6AP/caspase 8, which can then be used to design one or more highly specific inhibitors.

Overall, these results regarding the combination of small molecules with TRAIL/chemotherapeutic agents suggest a promising and novel therapeutic approach for the effective and selective killing of HPV^+^ cancer cells and may provide the basis for developing an effective therapeutic strategy to treat HPV-mediated cancers.

## Materials and Methods

### Protein purification

The construction of the pGEX-E6, pTriEx-E6AP, and pTriEx-Caspase-8 DED plasmids has been reported.^33^ Expression and purification of GSTE6, His-E6AP, and His-Caspase-8 DED were carried out as previously described.^33, 37^ GST-tagged and His-tagged proteins were diluted into GST dilution buffer (PBS pH 8.0, 5% glycerol, 2 mM DTT) and His dilution buffer (20 mM Hepes pH 7.4, 150 mM NaCl, 2 mMKCl, 5% glycerol, 2 mM DTT), respectively. Protein concentration was measured using Coomassie Plus – The Better Bradford Assay Reagent (Thermo Scientific, Waltham, MA, USA). Purity of the isolated proteins was estimated following separation by sodium dodecyl sulfate-polyacrylamide gel electrophoresis (SDS-PAGE) and Coomassie staining.

### Small-molecule library and acquisition of additional compounds

The 2000-compound small-molecule library (ActiProbe 2K) was acquired from TimTec, LLC (Newark, DE, USA) and was chosen because it encompasses a highly diverse selection of lead-like compounds. Five additional derivatives were purchased from Sigma (St. Louis, MO, USA) (benzimidazole, 2-(methoxymethyl)-1H-benzimidazole, and 3-(1H-Indol-1-yl)propan-1-amine methanesulfonate) and TimTec (1H-benzimidazole-1-methanol and 4,5,6,7-tetrahydro-1H-imidazo[4,5–c]pyridine-6-carboxylic acid). (Note: The material provided by TimTec as 6,7-dihydro-1H-imidazo[4,5–c]pyridine-6-carboxylic acid was analyzed by ^1^H-NMR and MS and found to be instead 4,5,6,7-tetrahydro-1H-imidazo[4,5–c]pyridine-6-carboxylic acid whose common name is spinacine. Furthermore, comparison with the spectra of authentic spinacine (purchased and synthesized) confirms this identification.)

### Screening of the small-molecular library

AlphaScreen Technology was used to assess the interactions between GST-E6, GST-caspase 8, His-E6AP, and His-caspase 8. Binding assays were performed in white 384-well plates (Perkin-Elmer) in a total volume of 25 *μ*l as previously described.^33^ Briefly, 5 *μ*l (50 ng) of GST-E6 and 5 *μ*l (87.5 ng) of His-caspase 8 were included in each reaction mixture with 5 *μ*l blocking buffer (0.5 mg BSA, 0.5% Tween 20 in PBS) in the absence or presence of 10 *μ*M of each test chemical. Members of the library were present at 10 *μ*M in DMSO. After a 1-h incubation of the mixture at room temperature, 5 *μ*l donor beads and 5 *μ*l acceptor beads (Perkin-Elmer) were added to each well according to the manufacturer's protocol. The mixture was incubated in the dark at room temperature overnight, and the emitted signal was detected using the Envision Multilabel plate reader (Perkin-Elmer). In the presence of test chemicals, the binding affinity was calculated as a percentage of the binding in the presence of carrier only (DMSO).

### Cell culture

U2OS, SiHa, and C33A cells were obtained from the America Type Culture Collection (Manassas, VA, USA) and cultured in Eagle's minimal essential medium (Invitrogen, Carlsbad, CA, USA) supplemented as described previously.^33^ HNSCC cell lines were obtained from several sources: UM-SCC47-TC-Clone 3 (#47CL3), UD-SCC2-TC-Clone 5(#2TC), UPCI-SCC90-UP-Clone 35 (#90), and SCC 84 were a gift from Dr. John Lee, Sanford Research (South Dakota, USA). UMSCC 47 (#47), UMSCC 29 (#29), and UMSCC 104 (#104) were a gift from Dr. Thomas Carey, University of Michigan (Michigan, USA). HNSCC cells were cultured in Dulbecco's Modified Eagle Medium (Mediatech, Manassas, VA, USA) supplemented with 10% of FBS.

### Cell viability assay

The extracellular domain of human TRAIL was cloned into a pTriEx expression plasmid containing N-terminal Hisx6 tag. His-TRAIL was expressed in the *E. coli* BL-21 pLys strain and purified as previously described. Doxorubicin and cisplatin were purchased from Sigma. His-TRAIL, doxorubicin, and cisplatin were diluted in PBS to the desired concentration before using. To measure cell survival following treatment with TRAIL, cisplatin and doxorubicin, SiHa (2 × 10^4^/well), C33A (1 × 10^4^/well), and HNSCC cells (2 × 10^4^/well) were seeded into 96-well plates and allowed to adhere overnight. Small molecules at the desired concentration were added and incubated at 37 °C for 4 h. As indicated, TRAIL, cisplatin, or doxorubicin was then added. The TRAIL treatment group contained cycloheximide (5 *μ*g/ml) to inhibit *de novo* protein synthesis, and the cells were incubated for 16 h prior to measuring cell viability by the MTT assay preformed as described previously.^38^ All experiments were repeated at least three times (three biological replicates, carried out on different days), with each experimental group measured in triplicate within each of these individual experiments. Data presented are from a representative experiment.

### Caspase activity assay

Cells were plated into 96-well plates at a density of 2 × 10^4^ cells per well and incubated overnight. Small molecules were then added and incubated at 37 °C for 4 h. TRAIL (100 ng/ml), cisplatin (50 *μ*M), and doxorubicin (2 *μ*M) were then added. Cycloheximide (5 *μ*g/ml) was added along with TRAIL at the indicated time points. Caspase 3/7 activity was measured using the flourogenic substrate CellTitier-Glo for caspase 3/7 activity (Promega, Fitchburg, WI, USA) following the manufacturer's instructions. Briefly, cells were lysed by the addition of 20 *μ*l of 5 × passive lysis buffer (Promega). The plate was put on an orbital shaker and incubated for 10 min at room temperature. In all, 20 *μ*l of cell lysates were transferred to white plates, and either substrate alone or substrate plus the caspase 3/7 inhibitor was added to the appropriate wells. After a 10-min incubation, the released fluorophore was measured using a plate-reading fluorimeter (Flx800, Bio-Tek Instrument Co., Winooski, VT, USA). The activity in wells treated with inhibitor was subtracted from the activity in wells lacking inhibitor. The resulting difference was expressed as a percentage of the caspase activity of the untreated cells.

### Immunoblot analysis

Cells (1 × 10^6^) were collected and washed with PBS. All liquid was removed by centrifugation at 3000 r.p.m. for 5 min at 4 °C. The cell pellet was lysed in 100 *μ*l lysis buffer (50 mM Tris-HCl, pH 7.4, 150 mM NaCl, 1% NP-40, 1 mM EDTA, 5% glycerol, 1 mM dithiothreitol, 1 mM phenylmethylsulfonyl fluoride, with one tablet of protease inhibitor mixture (Roche Molecular Biochemicals, Basel, Switzerland) per 10 ml of buffer added just prior to use) for 10 min on ice. Lysates (10–40 *μ*g total protein/lane) were then subjected to 10 or 12% SDS-PAGE and transferred to PVDF membranes using the iBlot dry blotting system (Invitrogen). Anti-caspase 8 monoclonal antibodies (BD Pharmingen, Franklin, NJ, USA) and anti-*β*-actin monoclonal antibodies (Sigma) were applied at 1 : 5000 dilutions. The secondary antibodies used were goat anti-mouse IRDye800 (LI-COR Biosciences, Lincoln, NE, USA) for caspase 8 and goat anti-mouse Dy680 (LI-COR Biosciences) for *β*-actin at 1 : 30 000 dilutions. Signals were measured using the Odyssey Infrared Imaging system (LI-COR Biosciences) and expressed in relative light units.

### p53 ELISA

The p53 ELISA was performed as described previously,^26^ with some modifications. Antibodies secreted by clone pAb122 (hybridoma obtained from ATCC, antibodies purified from the culture medium using protein-A Sepharose) were used as monoclonal capture antibodies. This antibody has a broad specificity, binding to both human and mouse forms of p53 and to both wild-type and mutant forms of the protein. These antibodies were absorbed (50 *μ*l per well, 4 *μ*g/ml) onto the surfaces of a Nunc-immuno plate, MaxiSorp Surface (NalgeNunc International, Rochester, NY, USA) by incubation overnight at 4 °C. The plates were then washed on an Auto Strip Washer, EL_X_50 (Bio-Tek Instruments, Inc.) using PBST (PBS plus 0.1% Tween 20) six times. Nonspecific binding sites were blocked by incubation with 200 *μ*l per well of PBS, including 10% calf serum (Invitrogen) (blocking buffer) for 2 h at room temperature, followed by washing as described above. In all, 100 *μ*l of each cell lysate to be tested were then added to the coated wells and allowed to incubate overnight at 4 °C. After washing, 100 *μ*l of a solution containing 4 *μ*g/ml biotinylated anti-p53 antibodies (polyclonal, produced in sheep, Roche) diluted into blocking buffer was added to each well and allowed to incubate for 45 min at room temperature. The plates were washed, and then the avidin–peroxidase conjugate (Sigma; 100 *μ*l/well, 2.5 *μ*g/ml diluted into blocking buffer) was added and allowed to incubate for 30 min at room temperature. After washing, 100 *μ*l of the substrate (0.3 mg/ml ABTS (2,2'-azino-di-(3-ethylbenzthiazolin sulfonate) dissolved into 0.1 M citric acid, pH 4.35, with 1 *μ*l/ml 30% H_2_O_2_ added just before use) was added to each well and allowed to incubate for approximately 30 min. The absorbance at 405 nm was read with a microplate reader (Dynex Technologies; MRX Revelation software, Chantilly, VA, USA). The protein concentration of each lysate was also measured using the BCA method (Pierce, Chantilly, VA, USA) and used to normalize the measured p53 values for possible variations in the number of cells per well. Each p53 value (obtained from the ELISA assay) was divided by the protein concentration to obtain a normalized p53 value (ng p53 per mg total protein). The average and S.D. of the replicates (a minimum of three) were calculated, normalized to the control, and used to prepare the graph.

## Figures and Tables

**Table 1:** EC_50_ (*μ*M) values of tested small molecules for the indicated protein–protein interactions

	(**1**) [*μ*M]	**(****2**) [*μ*M]	(**3**) [*μ*M]	(**4**) [*μ*M]	Spinacine (**5**) [*μ*M]
GST-E6/His-caspase 8	0.63	1.038	No Inhibition	No inhibition	0.017
GST-E6/His-E6AP	0.67	1.481	No Inhibition	No inhibition	0.02
GST-caspase 8/His-caspase 8	0.83	1.051	No Inhibition	No inhibition	No Inhibition

**Table 2:** EC_50_ (*μ*M) values of the indicated compounds for cell toxicity and for TRAIL-induced apoptosis

	Myricetin [*μ*M]	(**3**) [*μ*M]	Spinacine (**5**) [*μ*M]
Cell toxicity	539.1	284.9	268.5
TRAIL-induced apoptosis	154.8	No sensitization	31.3

## Abbreviations

HPV: human papillomavirus
E6AP: E6-associated protein
TRAIL: tumor necrosis factor-related apoptosis-inducing ligand
FADD: Fas-associated protein with death domain
HNSCC: head and neck squamous cell carcinoma
